# Simultaneous host and parasite expression profiling identifies tissue-specific transcriptional programs associated with susceptibility or resistance to experimental cerebral malaria

**DOI:** 10.1186/1471-2164-7-295

**Published:** 2006-11-22

**Authors:** Fiona E Lovegrove, Lourdes Peña-Castillo, Naveed Mohammad, W Conrad Liles, Timothy R Hughes, Kevin C Kain

**Affiliations:** 1 Institute of Medical Science, Department of Medicine, University of Toronto, Toronto, ON, Canada; 2 Center for Cellular and Biomolecular Research, University of Toronto, Toronto, ON, Canada; 3 Department of Medical Genetics and Microbiology, University of Toronto, Toronto, ON, Canada; 4 McLaughlin-Rotman Centre, McLaughlin Centre for Molecular Medicine, UHN and University of Toronto, Toronto, ON, Canada

## Abstract

**Background:**

The development and outcome of cerebral malaria (CM) reflects a complex interplay between parasite-expressed virulence factors and host response to infection. The murine CM model, *Plasmodium berghei *ANKA (PbA), which simulates many of the features of human CM, provides an excellent system to study this host/parasite interface. We designed "combination" microarrays that concurrently detect genome-wide transcripts of both PbA and mouse, and examined parasite and host transcriptional programs during infection of CM-susceptible (C57BL/6) and CM-resistant (BALB/c) mice.

**Results:**

Analysis of expression data from brain, lung, liver, and spleen of PbA infected mice showed that both host and parasite gene expression can be examined using a single microarray, and parasite transcripts can be detected within whole organs at a time when peripheral blood parasitemia is low. Parasites display a unique transcriptional signature in each tissue, and lung appears to be a large reservoir for metabolically active parasites. In comparisons of susceptible versus resistant animals, both host and parasite display distinct, organ-specific transcriptional profiles. Differentially expressed mouse genes were related to humoral immune response, complement activation, or cell-cell interactions. PbA displayed differential expression of genes related to biosynthetic activities.

**Conclusion:**

These data show that host and parasite gene expression profiles can be simultaneously analysed using a single "combination" microarray, and that both the mouse and malaria parasite display distinct tissue- and strain-specific responses during infection. This technology facilitates the dissection of host-pathogen interactions in experimental cerebral malaria and could be extended to other disease models.

## Background

Cerebral malaria (CM) is a major cause of global morbidity and mortality. The molecular basis of this syndrome remains incompletely defined [1]; however, the development, severity and ultimate outcome of malaria infections are known to be influenced by genetic factors in both the host and the parasite [1-3]. Murine models have been informative in clarifying the molecular mechanisms underlying CM. *Plasmodium berghei *ANKA (PbA) infection of mice simulates many of the features of human CM [3] and infection of genetically defined inbred mice provides an opportunity to dissect the host response to infection. C57BL/6 (B6) and other susceptible mice [4-6] infected with PbA develop malaria-associated encephalopathy similar to CM in humans. In contrast, BALB/c mice do not develop encephalopathy, although they become infected and achieve similar levels of parasite density [7]. Host factors, including differential intensity and timing of pro- and anti-inflammatory cytokine responses to infection, have been implicated in susceptibility to CM in this model [3].

Parasite-dependent factors also influence disease outcome. PbA infection of B6 mice results in CM, while infection of the same host with the closely related parasite line *P. berghei *K173 does not [8]. By comparing these models, it has been postulated that parasite-dependent modulation of host immune responses may contribute to the pathogenesis of CM [9].

Genome-wide expression profiling is being increasingly applied to dissect the complex details of the host response to malaria infection [10-16]. Delahaye *et al*. identified candidate mouse resistance genes to PbA by profiling brain gene expression in resistant and susceptible mice [15]. Sexton *et al*. examined transcriptional responses in the spleens of B6 mice infected with PbA and reported gene expression patterns suggestive of suppressed erythropoiesis, up-regulated host glycolysis and an interferon-inducible target response [14]. Several studies have also analyzed parasite expression patterns *in vitro *and *in vivo *[17,18]; however, concurrent examination of the host-parasite interaction has only been used to analyze the vector-parasite relationship between *Anopheles stephensi *and *P. berghei *[19].

Malaria infections result in diverse clinical outcomes presumably because of the dynamic relationship between parasite-expressed virulence factors and individual host response to these determinants. The objective of this study was to simultaneously examine both sides of the parasite-host interface to identify corresponding PbA and murine organ-specific expression profiles associated with resistance or susceptibility to CM. We used custom-designed "combination" microarrays containing both murine and plasmodial genes to define host and parasite transcriptional programs in target organs during PbA infection of CM-susceptible (B6) and resistant (BALB/c) mice. Malaria gene expression was readily detected within host tissue, and differential expression of parasite and murine genes discriminated infection in resistant from susceptible mice, identifying both host and parasite transcriptional programs which may contribute to CM.

## Results and discussion

### A combination murine/*P. berghei *ANKA microarray

We designed and tested a microarray (the "combination array") composed of 42,034 sixty-mer probes designed to detect over 20,000 known and predicted mouse transcripts [20] as well as 17,313 known or predicted *P. berghei *genes and ESTs. Because both the mouse and malaria genomes are still being annotated, and the statistical analyses employed are sensitive to artefacts that can arise from duplicated genes, and inclusion of pseudogenes and unverified genes, we only considered probes in our computational analyses that uniquely target 9,035 single-copy mouse genes and 8,577 independent *P. berghei *genes or ESTs (henceforth referred to collectively as "genes") annotated in current genome databases.

**Figure 1:**
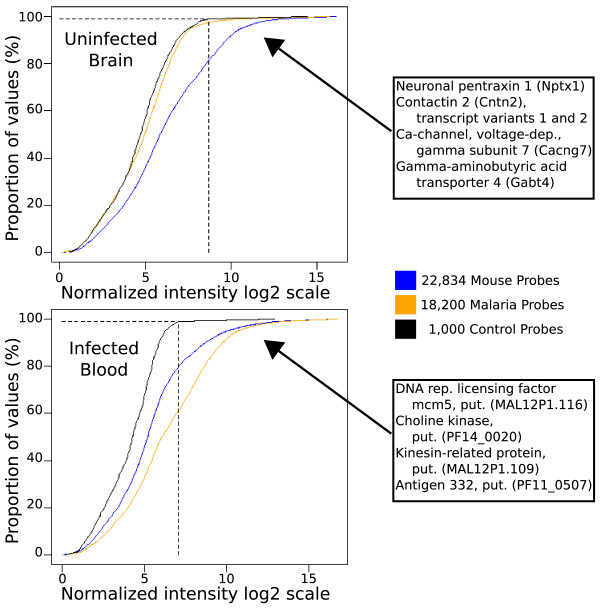
**Microarray probe specificity**. A test hybridization was performed using cDNA from uninfected mouse brain and from PbA-infected mouse blood. The curves show the cumulative distribution of normalized log2 intensity values for control (black), malaria (orange) and mouse (blue) probes. The dashed black lines indicate the 99^th ^percentile for the negative control probes. The top panel represents data from uninfected brain and shows that intensity values of malaria probes did not differ from the random-sequence control probes, indicating that mouse cDNA did not significantly cross-hybridize to malaria probes. The PbA-infected blood hybridization (bottom panel) demonstrated that PbA transcripts could be detected in the presence of mouse transcripts at standard hybridization conditions. Examples of top-expressed genes are given in the text boxes.

To test the specificity of our combination array, we hybridized one microarray with Cy3-labelled cDNA from uninfected B6 brain and Cy5-labelled cDNA from PbA infected B6 blood, containing approximately 20% parasitized red blood cells. In uninfected brain, the spot intensity distribution from malaria probes was indistinguishable from that of random-sequence negative control probes on the array, while 20% of the mouse probes exceeded the 99^th ^percentile of negative control probe intensities (Figure 1), presumably representing the fraction of genes expressed in brain. In contrast, 25% of the malaria probes exceeded the 99^th ^percentile of negative control probe intensities in infected blood. This indicates that the malaria probes were functional under our standard hybridization conditions and had minimal cross-hybridization to mouse cDNAs. Therefore, the combination array was able to specifically detect parasite and host gene expression.

### Host and parasite tissue-specific expression patterns

**Figure 2:**
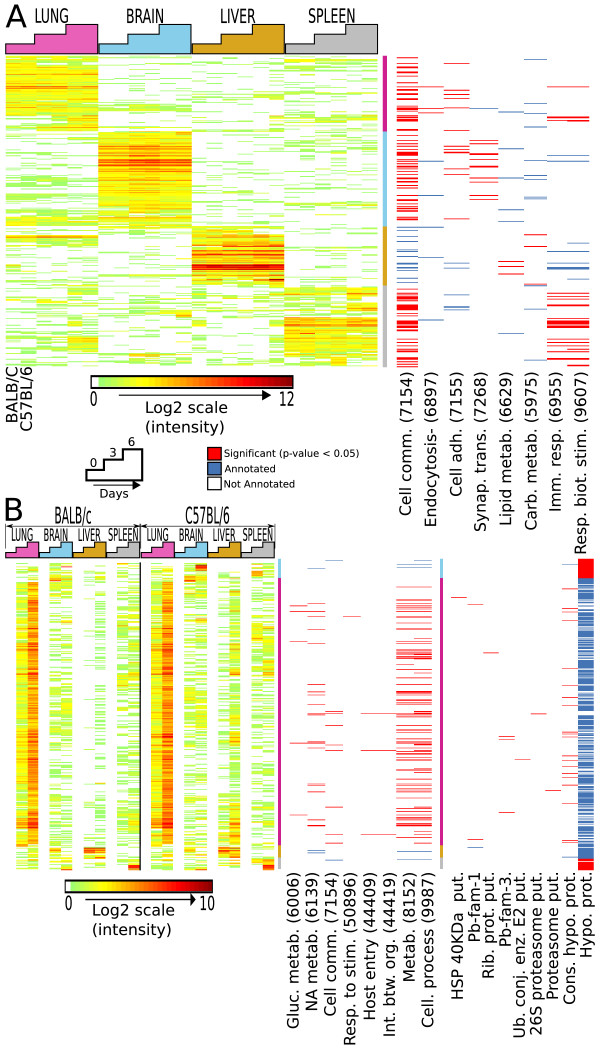
**Overview of mouse and malaria gene expression in all organs**. **A. **Highly expressed mouse genes. 2268 mouse genes were expressed in the top 25th percentile. Left, normalized intensity values of genes (in rows) are ordered using Pearson correlation and hierarchical clustering. Samples (columns) are grouped chronologically (i.e. Day 0, 3, and 6) by organ. Right, GO-gram indicates statistically significant enrichments of select GO-BP functional annotations in gene clusters. Red bars indicate that a particular gene is annotated with the given GO-BP category and that the GO-BP category is significantly over-represented within the cluster in which the gene was placed. Blue bars indicate that a particular gene is annotated with the given GO category and that the GO category is not significantly enriched within the given cluster. Not all GO categories that displayed significant enrichment are shown. For brevity, GO-BP category names are abbreviated and final digits of GO identifiers are shown in parentheses. The vertical multi-colored bar denotes gene clusters (pink indicates genes highly expressed in the lung, blue in brain, yellow in liver and grey in spleen). GO-BP categories overrepresented in each of the known organs correlate with known function, e.g. GO-BP categories significantly enriched in the spleen cluster include "immune response" and "response to biotic stimulus". **B. **Highly expressed malaria genes. 2159 malaria genes were expressed in the top 25^th ^percentile. Left, normalized intensity values of genes were ordered using Pearson correlation and hierarchical clustering. Samples are grouped chronologically by organ. Middle and Right, GO- and Protein-gram indicate statistically significant enrichments of GO-BP or Pfam annotations in highlighted gene clusters. Color codes are as in A. Interestingly, the majority of highly expressed PbA genes were found in the lung and functional annotations for these transcripts included categories related to metabolism or host-parasite interaction.

We next hybridized combination arrays with cDNA from four malaria target organs (lung, brain, liver, and spleen) from B6 (susceptible) and BALB/c (resistant) mice over a time-course of infection (pre-infection [day 0], at an early time point with low parasitemia [day 3] and just prior to the development of CM in susceptible mice with high parasitemia [day 6]). In total, we detected 2,829 mouse genes with expression above the 99^th ^percentile of negative-control probes in at least one tissue, among which 2,268 were detected at least 6-fold higher in at least one tissue relative to the gene median expression across all tissues (Figure 2A). We confirmed that these transcripts displayed tissue-specific gene expression patterns similar to those previously described [20], including enrichment of expected Gene Ontology Biological Process (GO-BP) categories [21] among the genes expressed in different tissues (Figure 2A). For example, genes highly expressed in the spleen were significantly associated with the GO-BP categories of 'Immune Response' and 'Response to Biotic Stimulus'.

We detected 5,632 malaria genes with expression above the 99^th ^percentile of negative-control probes in at least one tissue, among which 2,159 were detected at least 15-fold higher in at least one infected tissue relative to uninfected tissue. This indicated that they were unlikely to represent cross-hybridization to mouse transcripts in the same samples and were roughly consistent with the number of malaria genes previously found to be expressed in infected blood [17,22]. PbA transcripts also displayed an organ-specific "signature" of gene expression that was modulated over the course of infection (Figure 2B).

Strikingly, the majority of PbA transcripts with detected expression were present in the lung (Figure 2B). Although information about the PbA genes is sparse, the lung-expressed genes encoded apparent heat shock proteins, ribosomal proteins, and proteasome components, and the cluster is significantly enriched with GO-BP annotations of nucleic acid metabolism, entry into host, and metabolism (P < 0.05). Further analysis of the lung-expressed genes, using *P. falciparum *Protein-Protein Interaction (PPI) sub-networks identified by LaCount *et al*. [23], showed that a number of gene pairs in the PPI network were significantly over-represented in the group of PbA genes expressed in the lung (P < 0.001), including some associated with cell invasion (P < 0.05) [See Additional file 1]. These findings suggest that the lung may be a preferential site of PbA biosynthesis, metabolism and proliferation. Although this intriguing observation may reflect, at least in part, pulmonary blood volume, models using PbA in mice [24] and *P. falciparum *in rats [25] indicate that parasites do sequester in the lung. Examination of parasite transcription profiles shows that parasites are undergoing replication and metabolic functions in the lung and are displaying activity other than simple adhesion. Parasite sequestration and replication in the lung would be expected to induce the release of parasite products, cytokine production, and recruitment of immune cells, thereby causing pulmonary inflammation and/or injury. Indeed pulmonary pathology has been reported in previous studies of PbA infection, including increased pulmonary vascular permeability and edema [8,26-28]. Additionally, respiratory distress is associated with severe malaria due to *P. falciparum *[29,30], and pulmonary edema often occurs in individuals who develop CM [31,32].

### Baseline differences and unique temporal responses to PbA infection in CM-resistant (BALB/c) versus CM-susceptible (B6) mice are predominantly associated with immune function

**Figure 3:**
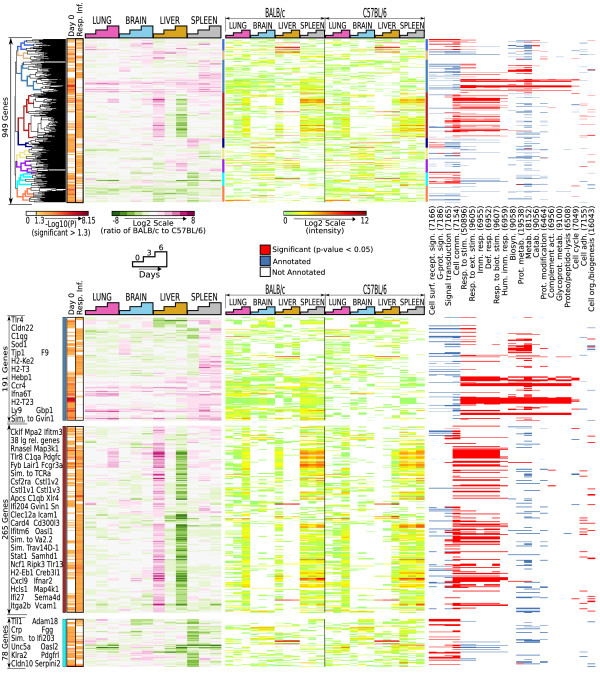
**Differentially expressed genes at baseline (before infection) and over the infection time course between resistant (BALB/c) and susceptible (B6) mice**. 608 mouse genes were differentially expressed prior to infection and 607 mouse genes were differentially expressed over infection (linear model, *P *< 0.05), with 266 differentially expressed in both categories. Left, heat maps showing significance levels at day 0 or responding to infection (Resp. Inf.), which shows whether differentially expressed genes were significantly different prior to infection, over infection or both. Middle left, log2-scale ratios of BALB/c to B6 intensity values of the 949 differentially expressed genes (in rows) grouped using cosine-angle correlation and hierarchical clustering. Samples (columns) are chronologically ordered by organ. Middle, corresponding intensities for each gene (normalized and median-subtracted) and for both mouse strains are shown. Nine major clusters are highlighted on the dendrogram with a vertical, multi-colored bar. Right, GO-gram indicating statistically significant enrichments of functional (GO-BP) annotations in the highlighted clusters. Numbers in parenthesis correspond to final digits of GO identifiers. Red bars indicate that a particular gene is annotated with the given GO-BP category and that the GO-BP category is significantly over-represented within the cluster in which the gene was placed. Blue bars indicate that a particular gene is annotated with the given GO category and that the GO category is not significantly enriched within the given cluster. Not all GO categories that displayed significant enrichment are shown. Lower panel, detailed view of representative clusters including highlighted genes of interest. Clusters of differentially expressed genes were up-regulated in resistant mice at day 0 and remained up-regulated over infection (e.g. "blue" cluster), changed over infection (orange) or were up-regulated in susceptible mice at day 0 and remained up-regulated over infection (turquoise).

Different clinical outcomes to malaria may be attributable to the differential immunological resting state of resistant (BALB/c) and susceptible (B6) hosts – that is, differences in transcriptional status at baseline may influence the subsequent course of infection. Additionally, host response during infection is likely to contribute to outcome. To test these hypotheses, a linear model was employed to compare expression profiles in specific tissues of both mouse strains at baseline (day 0) and between baseline and post-infection (days 3 and 6 versus day 0). Six hundred and eight (608) mouse genes were expressed differentially at baseline (P < 0.05), and 607 genes showed a significant differential response to infection (P < 0.05) between resistant and susceptible mice. Two hundred and sixty-six (266) genes were differentially expressed both at baseline and over infection, and 949 genes were differentially expressed at baseline and/or during the course of infection. To further characterize these 949 genes, we performed clustering analysis of gene expression at each time point in all tissues (Figure 3). This revealed several groups of genes with distinctive profiles over the time course, many of which are enriched for immunological GO-BP categories.

The expression of one group of 191 genes was consistently greater in resistant mice at baseline and over infection (Figure 3 lower panel, metallic blue bar). Functional categories significantly enriched in this group included 'defence response', 'immune response', 'complement activation' and 'humoral immune response'. Immune-related genes within these groups included interferon-inducible guanylate nucleotide binding protein 1 (*gbp1*) and chemokine (C-C motif) receptor 4 (*ccr4*).

Of interest was a cluster of 265 genes with significant enrichment in immune related GO categories. This cluster was highly expressed at baseline in livers of resistant animals, up-regulated in livers of susceptible mice over the course of infection, as well as being upregulated in lungs and spleens of resistant mice during infection (Figure 3 lower panel, maroon bar). Functional annotations associated with this cluster included 'cell adhesion', 'cell communication', 'response to stimulus', 'external stimulus and biotic stimulus', and 'defence, immune and humoral immune response'. Several immunologic cell surface molecules were clustered within this group (e.g., *vcam1*), as were numerous genes containing immunoglobulin (Ig) domains. Additionally, 38 transcripts encoded or putatively encoded Ig family member genes. Other potentially important transcripts figure included cytokine receptors, 2 subcomponents of complement component 1q, at least 9 interferon-inducible genes and several signalling molecule transcripts.

A third gene cluster of interest (Figure 3 lower panel, turquoise bar), significantly associated with the GO annotations of 'signal transduction', 'cell communication', 'cell surface receptor linked signal transduction' and 'G-protein coupled receptor signalling pathway', was up-regulated in susceptible mice at baseline and in response to infection. Genes encoding proteins likely involved in cell communication included platelet-derived growth factor receptor-like (*pdgfr1*) and members of the *uPAR/Ly6/CD59 *family. C-reative protein (*crp*) was also highly up-regulated in the lungs of resistant mice at baseline, decreasing over infection.

Additional analysis of the genes selected by the linear model, using the Ingenuity pathways analysis program, identified pathways and gene networks enriched in the expression data and therefore likely to be important in CM pathogenesis. Pathways significantly associated with all genes selected by the linear model (right-tailed Fisher's Exact Test, p < 0.05) include pyruvate metabolism, p38 MAPK signalling and platelet-derived growth factor (PDGF) signalling [see Additional file 2]. Pyruvate kinase deficiency has been shown to protect animals from severe disease in another murine malaria model, *P. chabaudi chabaudi *[33]. *In vitro *studies have demonstrated that *P. falciparum *glycosylphosphatidylinositol (GPI) moities stimulate macrophages to produce the proinflammatory cytokines IL-6 and IL-12 via a p38 MAPK-dependent pathway [34]. Although no studies have directly linked PDGF signaling with malaria infection, PDGF is known to stimulate proliferation and chemotaxis in many cell types, including leukocytes; Moreover, platelets are a source of PDGF and may be important in PbA pathogenesis [5,35].

**Figure 4:**
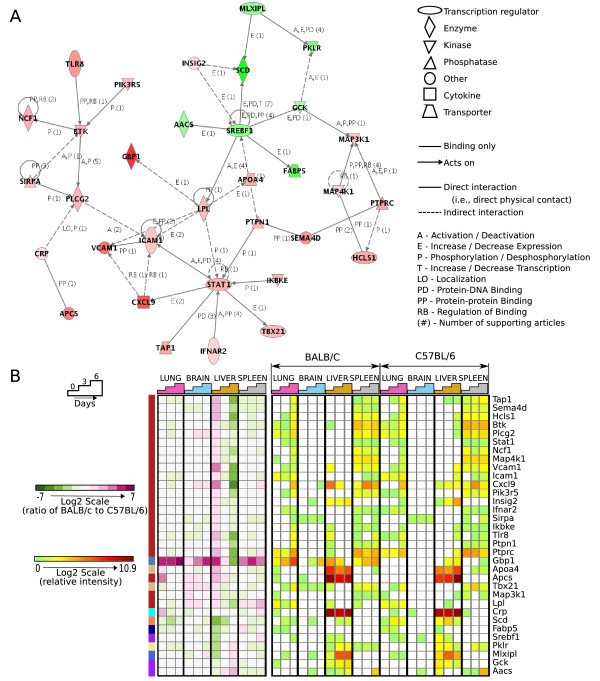
**A network involving genes differentially expressed at Day0 may be associated with protective early immune response to infection**. **A. **Genes differentially expressed at baseline in liver were found to be significantly overrepresented in this network by the Ingenuity Pathway Analysis software (*P *< 0.05). This network involves the transcription factor *stat1 *and many other immunologically important transcripts encoding cell adhesion molecules (*icam1*, *vcam1*), receptors (*tlr8*,*ifnar2*) acute phase proteins (*crp*, *apcs*) and others (*cxcl9*, *gbp1*, *ikbke*). Increasing colour intensities indicate relative up-regulation of gene expression in resistant (red nodes) compared to susceptible mice (green nodes). **B. **Clustergram showing log2-scale ratios of BALB/c to B6 intensity values of the genes found in the network (A). Genes (in rows) are grouped using cosine-angle correlation and hierarchical clustering, and samples (columns) are chronologically ordered by organ. Many of these genes which were upregulated in BALB/c liver at baseline become up-regulated in susceptible (B6) mice over the course of infection.

Analysis of genes differentially expressed in the liver at day 0 identified a network involving several immune-related transcripts, including signal transducer and activator of transcription 1 (*stat1*), a transcription factor involved in several inflammatory signaling processes (Figure 4A). Many genes in this network were highly expressed in resistant mice at baseline and became up-regulated in susceptible mice over the course of infection (Figure 4B). Therefore, Stat1-mediated signaling, which includes the interferon-α/β and interferon-γ signaling pathways, may moderate survival early in PbA infection. Our analysis shows that resistant BALB/c mice are primed for this response at baseline, while B6 mice show a delayed response, which may contribute to poor outcome. Both interferon-α/β and interferon-γ signaling have previously been associated with malaria infection. Development of CM in PbA infection requires both IFNγ and IFNγ receptor [28,36]. However, early production of IFNγ, as observed in the K173 murine model of non-cerebral malaria may protect against subsequent progression to cerebral malaria [9]. Moreover, IFNα and IFNγ receptor polymorphisms have been linked to protection from severe and cerebral malaria in humans [37,38]. Recent work comparing transcriptional responses in presymptomatic versus clinically apparent *P. falciparum *infection identified IFNγ signalling pathway members, including *STAT1*, to be induced early in infection [16], lending further support to the role of this signalling pathway. Since the differential response appears to involve a temporal sequence (i.e., early expression occurs in mice that survive acute infection and late expression involves CM development), enhancement of this pathway at appropriate times during the course of infection could improve the outcome of susceptible animals. Further experiments in a broader range of mouse strains will be required to examine how Stat1-mediated signalling, including early interferon-α/β and/or-γ mediated responses, modulates the progression of plasmodial infections and whether it could be used as a biomarker to predict resistance to CM.

### Malaria gene expression is modified by host genetic background and tissue microenvironment

**Figure 5:**
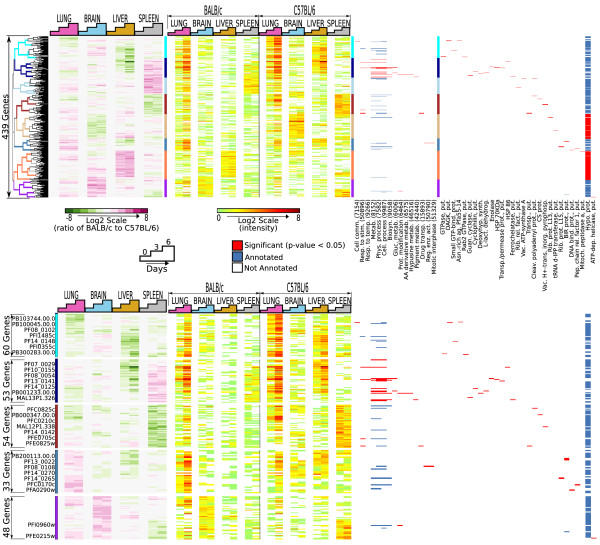
**Malaria genes differentially expressed across the infection time course in resistant and susceptible mice**. 439 PbA genes were selected as differentially expressed in resistant and susceptible hosts during infection (linear model, *P *< 0.05). Left, log2-ratios of BALB/c to B6 intensity measurements of PbA genes (rows) are clustered using cosine-angle correlation. Samples (in columns) are chronologically ordered by organ. Middle, intensities (normalized and median-subtracted) are shown for each gene in each mouse strain. Eight major clusters are highlighted on the dendrogram and using a vertical, multi-colored bar. Right, GO- and Protein-grams indicating statistically significant enrichments of GO-BP or Pfam annotations in the highlighted clusters. GO-BP and Pfam names are abbreviated and numbers in parenthesis correspond to final digits of GO identifiers. Red bars indicate that a particular gene is annotated with the given category and that the category is significantly over-represented within the cluster in which the gene was placed. Blue bars indicate that a particular gene is annotated with the given category and that the category is not significantly enriched within the given cluster. Not all GO and Pfam categories that displayed significant enrichment are shown. Lower panel, detailed view of representative clusters including highlighted genes of interest. The clusters identify gene groups that show remarkable differences in expression between hosts and also between tissues, giving evidence that PbA transcription is influenced both by the host response and tissue microenvironment.

We hypothesized that malaria gene expression would differ depending on the susceptibility status of the mammalian host. Therefore, we examined differential PbA gene expression between mouse strains. Using a linear model, 469 malaria genes were identified as differentially expressed (P < 0.05, Figure 5). Of note, when clustered, these genes showed striking differences, not only between resistant and susceptible hosts, but also between the target tissues examined (Figure 5).

An initial group of 60 genes, expressed in the lung and liver of susceptible (B6) mice (Figure 5 lower panel, turquoise bar) was over-represented in the GO category of 'cell communication'. One major cluster defined a set of 53 co-expressed genes highly up-regulated in the lungs and livers of susceptible (B6) mice and spleens of resistant (BALB/c) mice (Figure 5 lower panel, navy bar). GO categories significantly enriched in this cluster included 'response to stimulus and temperature', 'glucose, amino acid, hypusine and pigment metabolism' and the four predominant GO categories of 'biosynthesis', 'cellular process', 'metabolism' and 'physiological process'. The cluster representing 54 highly expressed genes in spleens of susceptible mice (Figure 5 lower panel, maroon bar) had limited functional annotations, but was significantly represented in the GO categories of 'response to stimulus' and 'drug transport'. A smaller cluster of 33 genes, which was up-regulated in the lungs of resistant mice and to a lesser extent in the livers of susceptible animals, was associated with regulation of enzyme activity and mitotic interphase (Figure 5 lower panel, light blue bar). Lastly, a prominent cluster containing predominantly putative genes lacking GO annotations appeared in the brains of resistant mice (Figure 5 lower panel, purple bar).

Although it was anticipated that parasites might respond differently in resistant versus susceptible hosts, the unique transcriptional signature that the parasite displayed in each of the four organs examined provides *in vivo *evidence that malaria parasites are profoundly influenced by the host genetic background and tissue microenvironment in which they replicate or sequester. The systematic transcriptional profiling of the same parasite isolate in multiple organs identified differential expression of a number of enzymes involved with energy pathways, cell signalling molecules, and genes encoding heat-shock proteins, some of which may constitute the parasites' own response to host defence. However, many of the transcripts remain annotated as hypothetical proteins and several evident gene clusters, especially those transcribed in the brains, have no gene ontology or protein information associated with them. Regardless, further investigation of these differentially expressed clusters may yield new potential targets for drug development and provide further insights into the regulation of plasmodial virulence determinants *in vivo*.

### Verification of expression analysis using quantitative real-time RT-PCR

Quantitative real-time RT-PCR (qRT-PCR) was performed to analyze the expression of a number of representative mouse genes (including cell adhesion molecules, cytokines, and interferon-inducible transcripts) and PbA genes, which were either identified by our statistical analysis or previously shown to be important in other studies but not identified by our analysis (e.g. TNF-α). qRT-PCR results correlated well with normalized intensity data in each organ at each time point, yielding a median overall correlation of 0.724 with the microarray data, and thereby confirming the validity of observed gene expression patterns [see Additional file 3].

### Analysis of mouse and malaria gene groups previously associated with malaria disease pathogenesis

Previous studies in various experimental models of malaria have demonstrated that specific host responses may influence clinical outcome [14,15] and that certain malaria genes may contribute to parasite virulence or disease pathogenesis. Based on the results from these previous studies, we chose to examine expression patterns of genes represented on the array in specific categories associated with malaria infection. For mouse genes, functional categories included 'cell adhesion', 'toll-like receptors', 'immune response' (cytokines, chemokines and their receptors), 'interferon responsive genes', 'acute phase proteins', 'erythroid-associated transcripts', 'complement activation', 'glycolysis' and 'hemostasis' [see Additional file 4]. PbA transcripts examined included the *bir *family of genes, orthologues of the P. falciparum *rif/stevor *genes [39] which encode for variant surface antigens [40]; an orthologue of sequestrin and the GO-BP category of 'invasion' [see Additional file 5]. While many of the PbA transcripts were in the top quartile of expressed genes, especially in the lungs, none of the chosen groups showed a significant enrichment of genes selected by the linear model (hypergeometric distribution, P < 0.05) and only two were differentially expressed between resistant and susceptible animals. This finding suggests that while parasite surface molecules and invasion-associated genes may be important in infection, they are not associated with a parasite phenotype that promotes the development of CM.

**Figure 6:**
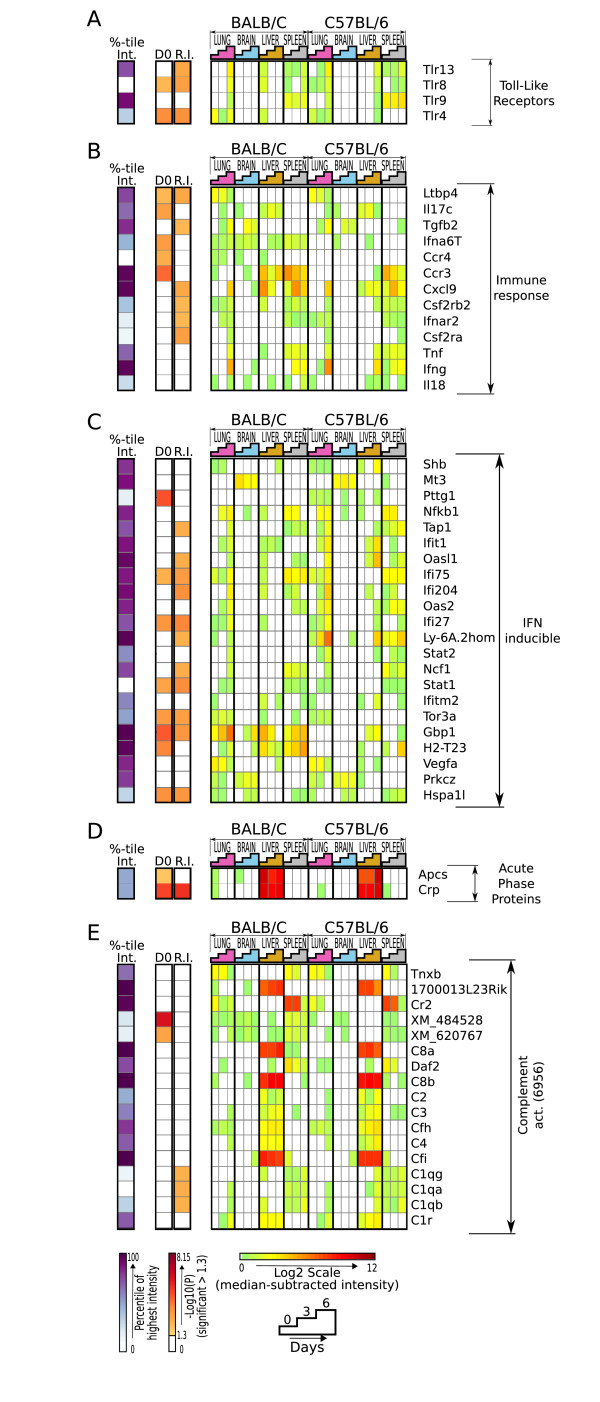
**Gene groups previously implicated in host response to malaria with significant enrichment of genes selected by the linear model**. The hypergeometric distribution was used to determine whether there was significant overlap between genes in categories previously implicated in malaria and those chosen by the linear model. Categories that showed an enrichment of differentially expressed genes included: **A. **Toll-like receptors, **B. **Immune response (cytokines, chemokines and their receptors), **C. **Interferon responsive genes, **D. **Acute phase proteins and **E. **Complement activation. Intensity values (median-subtracted, normalized, log2 scale) are shown for mouse genes (rows) represented on the microarray, in functional categories previously associated with malaria infection. Samples (columns) are chronologically ordered by organ. Left, heat maps showing relative intensity (%-tile Int.), and significance levels at day 0 (D0) or responding to infection (R.I.), which shows whether the corresponding gene was expressed significantly different between resistant and susceptible mice prior to infection, over infection or both. The final digits of its GO identifiers in parenthesis indicate GO-BP categories. Genes in each group were ordered using cosine-angle correlation and hierarchical clustering on the log-ratios.

However, in several host gene groups, there was a significant enrichment of differentially expressed genes selected by the linear model (Figure 6). This emphasizes the role of toll-like receptors; cytokines, chemokines and their receptors; interferon responsive genes; acute phase proteins and complement activation groups in the PbA CM model. In our analysis, many IFN-inducible genes were identified as differentially expressed (Figure 6C), lending further support for the importance of the timing and magnitude of interferon responses in mediating outcomes in PbA infection. With this and other microarray studies [13,14,16] identifying the expression of interferon-inducible genes in response to *P. berghei*, it could be argued that differences in constitutive and early innate immune responses by IFN-regulated genes, could contribute to innate resistance in BALB/c mice, and may be prognostic of outcome.

This analysis also identified complement-related genes as a group likely to be important to PbA susceptibility (Figure 6E). Epidemiological evidence links decreased complement receptor 1 (CR1) expression with reduced malaria incidence and disease severity [41]. Additionally, increased expression of C1qbeta is found in the brains of susceptible mice, compared to resistant ones, in PbA infection [15]. As with unregulated cytokines, a defective or deregulated complement response may also contribute to the pathophysiology of malaria.

## Conclusion

In this study, we designed and applied a combination oligonucleotide microarray to simultaneously interrogate organ-specific transcriptional responses on both sides of the pathogen-host interface in experimental murine malaria. The use of this and other host and/or plasmodium microarrays to examine parasite transcriptional profiles *in vivo *[10-15,17], demonstrates important features of how both host and pathogen respond during infection. A host/parasite combination array not only offers the advantage of providing concurrent information about both organisms, but is also more time- and cost-effective, and does not provide additional challenges for data analysis than using separate platforms.

The combination microarray data generated by this study provide an initial understanding of the dynamic tissue-specific bi-directional interaction between pathogen and host in malaria. Several unique insights emerged from this study. From the pathogen perspective, the lung is an important site of parasite metabolism and proliferation and that parasite transcriptional differences occurred not only between different hosts, but also within different organ environments. The differential response between resistant and susceptible mice was primarily associated with immune function and our analysis provides evidence for the importance of interferon signalling, interferon-responsive genes and complement-related genes in CM pathogenesis. Ultimately, when combined with complementary genetic strategies including targeted gene disruption, or extended to other host-pathogen pairs including humans and *P. falciparum*, such work may provide further insights into the pathogenesis of CM and facilitate the design of novel interventions targeting both host and parasite gene networks to improve clinical outcome in malaria.

## Methods

### Mouse/PbA combination microarray design

The 21,626 mouse probes were identical to those in a previous study for which hybridization was detected in at least one of 55 mouse tissues analyzed [20]. 12,328 known and predicted *P. berghei *gene sequences were downloaded from the *Plasmodium falciparum *Genome Projects website at the Wellcome Trust Sanger Institute[42]. *P. berghei *EST (5,582) and GSS (5,482) sequences were gathered from the University of Florida *P. berghei *Genome Tag Sequences website in August 2004 [43]. Redundancy checks were performed using sequence alignments (BLAST) with an e-value threshold of e^-8^. From the University of Florida sequences, 4,520 GSS sequences were found to be unique from ESTs. From the Sanger set, 7,211 sequences were found to be unique from ESTs. All University of Florida EST sequences were included in the final sequence set which totaled 17,313 malaria sequences. Sixty-mer probes were designed using a protocol in which T_m_-balanced sequences beginning every 10 bases in each target gene are evaluated for secondary structure, repeat content, and uniqueness, and a single probe that best balances these parameters was selected. One or two probes were designed for each of the malaria sequences. Probe sequences were submitted to Agilent Technologies (Palo Alto CA) for array manufacture.

### Mice and parasites

Experiments were approved by and conducted according to the University of Toronto animal ethics guidelines. Male B6 and BALB/c mice, 6–8 weeks of age, were obtained from Charles River Laboratories (Senneville QC). Cryopreserved *P. berghei *ANKA (MR4, Manassas VA) was thawed and passaged through naïve B6 donor mice.

### Experimental design

On day 0, prior to infection, 5 mice from each strain were euthanized and served as a baseline control for all experimental mice. Immediately following euthanasia, whole blood was collected by direct cardiac puncture, and intact brains, livers, spleens and lungs were excised, snap-frozen in liquid nitrogen and stored at -80°C until use. 10 mice from each strain were infected with 1 × 10^6 ^freshly isolated *P. berghei *ANKA parasitized erythrocytes by intraperitoneal injection. Parasitemia was monitored daily using thin blood smears stained with modified Giemsa (Protocol Hema 3 Stain Set, Sigma, Oakville ON). Five mice from each strain were sacrificed at both day 3 and day 6 following infection. Blood and organs were harvested as above.

### RNA isolation

Total RNA was extracted by homogenizing organs in Trizol reagent (Invitrogen, Burlington ON) according to the manufacturer's instructions, and mRNA was purified as described previously [20,44]. Briefly, RNA samples were denatured and loaded onto a 0.25 ml Oligo-dT cellulose (NEB, Mississauga ON) column in 40 mM Tris pH7.5, 1 M NaCl, 2 mM EDTA and 0.2% SDS. The columns were washed, and RNA was eluted in TE. This process was repeated once. Eluted mRNA was ethanol precipitated, resuspended in dH20 and stored at -80°C until use. Integrity of RNA was assessed by formaldehyde agarose gel electrophoresis.

### cDNA labeling and microarray hybridization

cDNA was reverse-transcribed from 1–-2 g mRNA using Superscript II reverse μ transcriptase (Invitrogen) with 1 μg random nonamer primers and 0.25 g T18VN per μ reaction. The reaction mix contained a final concentration of 1X RT Buffer, 10 mM DTT, 0.5 mM each dNTP and 0.5 mM 5-(3-aminoallyl)-2'deoxyuridine-5'-triphosphate (AAdUTP, Sigma). Following the RT reaction, RNA was hydrolyzed using NaOH/EDTA and cDNA was purified using QIAquick PCR Purification columns (Qiagen, Mississauga ON), washed with 80% ethanol and eluted in water. Purified cDNA was coupled with N-hydroxysuccinimide esters of Cy3 or Cy5 (GE Lifesciences, Baie d'Urfe QC) in bicarbonate buffer, following the manufacturer's instructions. Dye reactions were quenched by adding hydroxylamine and labeled cDNAs were separated from free dye molecules using QIAquick columns. Cy3 and Cy5-labeled cDNA pairs and Agilent control spots were added to a final volume of 0.5 ml hybridization buffer (1 M NaCl, 0.5% sodium sarcosine, 50 mM methyl ethane sulfonate (MES), pH 6.5, 33% formamide and 40 g salmon sperm DNA (Invitrogen)). Hybridizations were performed in Agilent hybridization chambers at 42°C with rotation for 18–24 hours. Slides were washed in 6X SSPE, 0.005% sarcosine, followed by 0.06X SSPE, allowed to dry and scanned with a 4000A microarray scanner. TIFF images were quantified with GenePix (Axon Instruments, Union City CA). Each array was hybridized with two samples representing equivalent tissues at equivalent time points in the different mouse strains.

### Normalization

The median intensity measurements extracted from the GenePix files were spatially detrended [20]. We applied variance stabilizing normalization (VSN) [45] in Bioconductor [46] and transformed to log2 scale. To compensate for potential cross-hybridization of mouse mRNA to malaria probes, the day 0 intensity value of each probe was subtracted from the day 3 and 6 intensity values in the same tissue. The intensity scale data (shown in Figures 2, 3 and 4) was median-subtracted across tissues. Because most genes are not expressed in most tissues, this prevents the clustering algorithm from seizing on noise amplified in the log scale. After the cross-hybridization removal step and median subtraction, all negative values were set to zero. Finally, the ratios of BALB/c to C57BL/6 intensity measurements in log2 scale (henceforth referred to as log ratios) were obtained after loess smoothing using Bioconductor [46] of VSN-normalized arrays.

### Microarray probe annotation

Gene names were obtained by pairwise alignment to Refseq (m33 May 2005) and Ensembl (v31 May 2005) using megablast [47]. The corresponding official gene names to the RefSeq and Ensembl IDs were obtained from the Mouse Genome Informatics (MGI). GO-Biological Process (GO-BP) annotations for mouse probes were from Zhang et al. [20]. PbA probes were aligned using blat [48] to the gene sequences provided by the *P. berghei *genome project at the Sanger Institute downloaded in July 2005 [42]. Since, to the best of our knowledge, there is no *P. berghei *functional annotation data, we annotated PbA genes by their P. *falciparum *orthologues using homology tables were obtained from Hall *et al*. [18]. Among the 8,577 PbA genes in the arrays, 2,616 have a P. *falciparum *orthologous gene whose identifier starts with "PF" or "MAL". *P. falciparum *GO-BP annotations were obtained from the Gene Ontology database downloaded in May 2005 and PlasmoDB annotations released in October 2002 [49]. Only 813 out of the 2,616 *P. falciparum *genes with a homologous PbA gene in the arrays have a functional annotation. Pfam assignments available for 8,502 malaria genes in the arrays were downloaded from GeneDB on November 24th, 2005 [50]. Of these, only 1,106 or 13% have a product name other than "hypothetical protein".

### Statistical analysis

For statistical analysis, linear models for microarray data [51] were applied to the normalized log ratios using the Limma software package version 1.9.6 [52] in Bioconductor. Linear models are a statistical approach similar to ANOVA, which generalize both ANOVAs and linear regressions. B6 samples were taken as reference in the design matrix and two contrast matrices were used. The first contrast matrix was used to estimate the difference between susceptible and resistant mice at day 0. The second contrast matrix was used to estimate the difference in the response to the infection between susceptible and resistant mice (i.e., to compare the difference between the expression level at day 0 and the average expression level of day 3 and 6 between the two mouse strains). Design and contrast matrices are available upon request. P-values for the four tissues were combined in a p-value per probe using the F-distribution. F-pvalues of probes mapped to the same gene were combined using Fischer method. Genes with a combined F-p-value < 0.05 were considered differentially expressed. P-values were not corrected for multiple testing.

### Clustering

We obtained the log ratios per gene by averaging the log ratios of probes mapped to the same gene. Genes were clustered according to their log ratio using either Pearson (Figure 2) or cosine-angle correlation (Figures 3, 4, 5, 6), and hierarchical clustering functions available in Bioconductor and R. The intensity diagrams show the median-subtracted intensity per gene obtained by averaging the relative measurements of probes mapped to the same gene. Clusters were selected for functional/annotation analysis by partitioning the corresponding dendrogram at a fixed height (between 1.2 and 1.7). The exact height was manually determined for each dendrogram based on the visual homogeneity of the sub-clusters and their size.

### Functional analysis

GO annotations were up-propagated using the GO-graph available in the GO package version 1.8.2 in Bioconductor. Enrichment of functional (GO-BP) or protein (Pfam) annotations on each sub-cluster was scored using the hypergeometric distribution. Annotations with a hypergeometric p-value smaller than 0.05 were considered to be significantly over-represented in a sub-cluster. For visualization, similar significant GO categories were joined together under their most specific common ancestor.

### Network and pathway analysis

Networks and pathways involving genes selected by the linear model were identified using Ingenuity Pathway Analysis software [53]. The analysis was performed on all 949 genes differentially expressed at baseline and across the course of infection (Figure 3), and also on genes differentially expressed per tissue. Significance values were calculated using a right-tailed Fisher's Exact Test. The one-tailed version of Fisher's Exact Test is identical to the corresponding test based on the hypergeometric distribution.

### Comparison between the malaria transcriptome and interactome

To explore whether there is a relation between the malaria expression profiles obtained in this work and protein-protein interaction (PPI) data, we compared the PPI network of P. *falciparum *described by Lacount, DJ *et al*. [23] with the cluster graph shown in Figure 2. Enrichment of protein-protein interactions in clusters was determined by random permutation of gene labels in the interaction network. There are 216 interacting protein pairs belonging to the same cluster among the 602 P. *falciparum *genes in the PPI network with a PbA homologue in the array. This intersection is significantly higher than expected by chance (P-value < 0.0008) when compared with the intersection obtained with 10,000 random networks. To determine whether any of the clusters were enriched for interacting proteins from any of the sub-networks [23], we used the hypergeometric distribution and selected those sub-networks with a p-value < 0.05.

### Quantitative real-time RT-PCR (qPCR)

cDNA was synthesized from 0.5 μg of mRNA using Superscript II reverse transcriptase with Oligo (dT)_12–18 _primers (Invitrogen). Serial dilutions of mouse or PbA genomic DNA purified from blood (Qiagen) were used as standards. gDNA standards or cDNA were added to the qPCR reaction containing 1X Power Sybr Green Master Mix (Applied Biosystems, Streetsville ON) and 0.5 μM primers in a final volume of 10 μl. qPCR was performed using the ABI Prism^® ^7900HT Sequence Detection System (Applied Biosystems). PCR primer sequences are posted on the website (below). Copy numbers were normalized to 5 mouse housekeeping genes (mouse) [54] or to cDNA concentration (PbA). qRT-PCR data and microarray data (normalized intensities) were compared by calculating the overall correlation of all organs at all time points in both mouse strains for each gene.

### Supplementary information

Detailed protocols, probe sequences and mappings, gene annotations, all microarray data, and information about the microarray hybridizations are posted on the project website [55]. The microarray platform (GPL4220 [NCBI #15193528]) and data series (GSE5672 [NCBI tracking system #15195573]) have been submitted to the Gene Expression Omnibus (GEO).

## Abbreviations

CM: Cerebral Malaria

PbA: *Plasmodium berghei *ANKA

B6: C57BL/6 mouse strain

GO-BP:Gene Ontology Biological ProcessPPI: Protein-Protein Interaction

*stat1*: signal transducer and activator of transcription 1

qRT-PCR: quantitative real-time RT-PCR

## Authors' contributions

FEL carried out the animal studies, organ and RNA isolation, performed microarray hybridizations, qRT-PCR and prepared the manuscript. LP-C performed all data analysis including probe annotation, normalization and statistical/functional analysis; and prepared the figures. NM wrote the software used for probe design, designed microarray probes and performed microarray hybridizations. WCL assisted with data interpretation. TRH conceived the "combination array" concept, designed microarrays and contributed to study design and coordination. KCK conceived the study, and contributed to its design and coordination. All authors helped to draft and approved the final manuscript.

## Supplementary Material

Additional file 1 Significant protein-protein interaction networks associated with Figure 2B (PbA organ-specific gene expression clusters) Analysis of organ-specific clusters of expressed genes found in *P. falciparum *Protein-Protein Interaction (PPI) networks. Several gene pairs in the PPI network were significantly over-represented in lung-expressed PbA genes (pink cluster, P < 0.001), including some associated with cell invasion (P < 0.05). Click here for file

Additional file 2 Significant pathways enriched with genes selected by the linear model. Additional analysis of the genes selected by the linear model, using the Ingenuity pathways analysis program, identified pathways enriched in the expression data and therefore likely to be important in CM pathogenesis. Pathways shown are significantly associated (right-tailed Fisher's Exact Test, p < 0.05) with all selected genes, all genes and organ-specific genes differentially responding to infection, or all genes and organ-specific genes differentially expressed at baseline (Day 0). Click here for file

Additional file 3 Correlation between qRT-PCR results and microarray intensity data. R^2 ^values for each gene, comparing qRT-PCR results and microarray intensity data at all time points in all tissues and both mouse strains. Click here for file

Additional file 4 Microarray data for gene groups previously implicated in host response to malaria. Intensity values (median-subtracted, normalized, log2 scale) are shown for mouse genes (rows) represented on the microarray, in several functional categories thought to be associated with malaria infection. Samples (columns) are chronologically ordered by organ. Left, heat maps showing relative intensity (%-tile Int.), and significance levels at day 0 (D0) or responding to infection (R.I.), which shows whether the corresponding gene was expressed significantly different between resistant and susceptible mice prior to infection, over infection or both. Categories examined included cell adhesion, toll-like receptors, immune response (cytokines, chemokines and their receptors), interferon responsive genes, acute phase proteins, erythroid-associated transcripts, complement activation, glycolysis and hemostasis. The final digits of its GO identifiers in parenthesis indicate GO-BP categories. Genes in each group were ordered using cosine-angle correlation and hierarchical clustering on the log-ratios. The hypergeometric distribution was used to determine whether there was significant overlap between genes in these categories and those chosen by the linear model. This analysis supports the role of toll-like receptors; cytokines, chemokines and their receptors; interferon responsive genes; acute phase proteins and complement activation in the PbA CM model. Click here for file

Additional file 5 Microarray data for PbA gene groups potentially involved with malaria pathogenesis. Intensity measurements (median-subtracted, normalized, log2 scale) are shown for PbA genes (rows) represented on the microarray, for BIR-related transcripts (BIR; BIR protein; BIR protein, putative; BIR protein, pseudogene, putative), merozoite-related transcripts (merozoite capping protein-1, merozoite surface protein 8, merozoite surface protein 1, precursor), sequestrin and genes in the entry into host cell GO-BP category. Samples (columns) are chronologically ordered by organ. Left, heat maps showing relative intensity (%-tile Int.), and significance levels responding to infection (Resp. Inf.), which shows whether the corresponding gene was expressed significantly different between resistant and susceptible mice over infection. Click here for file
