# Supplementary material for: Simultaneous host and parasite expression profiling identifies tissue-specific transcriptional programs associated with susceptibility or resistance to experimental cerebral malaria
Source: BMC Genomics. 2006 Nov 22;7:295. doi: 10.1186/1471-2164-7-295 (PMC1664577; doi:10.1186/1471-2164-7-295)
Supplement: Additional file 1 — Significant protein-protein interaction networks associated with Figure 2B (PbA organ-specific gene expression clusters) Analysis of organ-specific clusters of expressed genes found in P. falciparum Protein-Protein Interaction (PPI) networks. Several gene pairs in the PPI network were significantly over-represented in lung-expressed PbA genes (pink cluster, P < 0.001), including some associated with cell invasion (P < 0.05). [file 1471-2164-7-295-S1.doc]

Table 1: Significant Protein-Protein Interaction Networks Associated with Figure 2B (PbA Organ-specific Gene Expression Clusters)

| **Main Protein in PPI Subnetwork (LaCount *et al.*)** | **Figure 2 Cluster** | **Number of Cluster Genes in PPI subnetwork** | **Total Number of Genes in PPI subnetwork** | **Pvalue** |
| --- | --- | --- | --- | --- |
| **High interconnectivity** | |  |  |  |
| *PF11_0168 | Pink (Lung) | 8 | 13 | 0.003 |
| PF11_0507 | Pink | 19 | 46 | 0.003 |
| PFC0760c | Pink | 9 | 18 | 0.010 |
| PFC0315c | Pink | 3 | 3 | 0.012 |
| PF10_0206 | Grey (Spleen) | 1 | 4 | 0.012 |
| PF11_0504 | Pink | 12 | 28 | 0.014 |
| PF08_0054 | Pink | 7 | 13 | 0.014 |
| PF07_0126 | Pink | 8 | 16 | 0.015 |
| PF07_0016 | Grey | 1 | 6 | 0.018 |
| MAL8P1.69 | Pink | 9 | 20 | 0.023 |
| PFL0350c | Pink | 8 | 17 | 0.023 |
| PFF0920c | Grey | 1 | 8 | 0.024 |
| PF08_0132 | Pink | 10 | 24 | 0.029 |
| PF11_0241 | Grey | 1 | 10 | 0.030 |
| PF14_0499 | Pink | 8 | 18 | 0.034 |
| PFD0080c | Pink | 8 | 18 | 0.034 |
| PF10_0254 | Blue (Brain) | 1 | 10 | 0.038 |
| MAL6P1.147 | Blue | 1 | 11 | 0.041 |
| PFL2335w | Blue | 1 | 11 | 0.041 |
| PF10_0075 | Grey | 1 | 14 | 0.042 |
| PFC0120w | Blue | 1 | 13 | 0.049 |
|  |  |  |  |  |
| High correlation with mRNA expression | | | |  |
| PF07_0081 | Blue | 1 | 3 | 0.011 |
| PF10_0206 | Grey | 1 | 4 | 0.012 |
| MAL13P1.193 | Orange (Liver) | 1 | 16 | 0.012 |
| PF08_0055 | Grey | 1 | 5 | 0.015 |
| PFC0350c | Grey | 1 | 5 | 0.015 |
| PFE0990w | Grey | 1 | 6 | 0.018 |
| PF07_0120 | Blue | 1 | 5 | 0.019 |
| PF14_0046 | Blue | 1 | 6 | 0.023 |
| PF11_0098 | Grey | 1 | 8 | 0.024 |
| PFI1610c | Pink | 10 | 24 | 0.029 |
| PF11_0098 | Blue | 1 | 8 | 0.030 |
| PF10_0254 | Blue | 1 | 10 | 0.038 |
| PFL2335w | Blue | 1 | 11 | 0.041 |
|  |  |  |  |  |
| Enrichment of proteins sharing GO annotations | | | |  |
| MAL7P1.170 | Pink | 10 | 18 | 0.002 |
| *PF11_0168 | Pink | 8 | 13 | 0.003 |
| PF11_0507 | Pink | 19 | 46 | 0.003 |
| MAL8P1.34 | Blue | 1 | 2 | 0.008 |
| PF10_0077 | Blue | 1 | 2 | 0.008 |
| MAL7P1.171 | Orange | 1 | 10 | 0.008 |
| MAL8P1.17 | Pink | 5 | 7 | 0.008 |
| PF14_0197 | Pink | 4 | 5 | 0.011 |
| PFC0920w | Blue | 1 | 3 | 0.011 |
| PF14_0517 | Pink | 3 | 3 | 0.012 |
| MAL6P1.61 | Grey | 1 | 4 | 0.012 |
| PF11_0504 | Pink | 12 | 28 | 0.014 |
| PF08_0054 | Pink | 7 | 13 | 0.014 |
| PFE0185c | Blue | 1 | 4 | 0.015 |
| PFC0350c | Grey | 1 | 5 | 0.015 |
| PFE0990w | Grey | 1 | 6 | 0.018 |
| PFB0310c | Blue | 1 | 5 | 0.019 |
| PFB0680w | Blue | 1 | 5 | 0.019 |
| MAL8P1.69 | Pink | 9 | 20 | 0.023 |
| MAL7P1.81 | Blue | 1 | 6 | 0.023 |
| PF10_0272 | Blue | 1 | 6 | 0.023 |
| PFI0875w | Pink | 8 | 17 | 0.023 |
| PFL0350c | Pink | 8 | 17 | 0.023 |
| PF11_0098 | Grey | 1 | 8 | 0.024 |
| PF11_0396 | Blue | 1 | 7 | 0.027 |
| PFB0760w | Blue | 1 | 7 | 0.027 |
| MAL7P1.166 | Pink | 4 | 6 | 0.027 |
| PF10_0219 | Pink | 4 | 6 | 0.027 |
| PF08_0132 | Pink | 10 | 24 | 0.029 |
| MAL13P1.63 | Blue | 1 | 8 | 0.030 |
| PF11_0098 | Blue | 1 | 8 | 0.030 |
| PF11_0241 | Grey | 1 | 10 | 0.030 |
| PFI1830c | Grey | 1 | 11 | 0.033 |
| PFD0080c | Pink | 8 | 18 | 0.034 |
| PF11_0165 | Blue | 1 | 9 | 0.034 |
| MAL6P1.147 | Blue | 1 | 11 | 0.041 |
| PFD0545w | Blue | 1 | 11 | 0.041 |
| PFE0770w | Blue | 1 | 11 | 0.041 |
| *PF13_0197 | Pink | 8 | 19 | 0.047 |
| PF11_0086 | Grey | 1 | 16 | 0.048 |
| PFC0155c | Grey | 1 | 16 | 0.048 |
| PFC0120w | Blue | 1 | 13 | 0.049 |
|  |  |  |  |  |
| Enrichment of proteins sharing protein domains | | | |  |
| MAL7P1.170 | Pink | 10 | 18 | 0.002 |
| PF14_0197 | Pink | 4 | 5 | 0.011 |
| PF08_0054 | Pink | 7 | 13 | 0.014 |
| PF10_0272 | Blue | 1 | 6 | 0.023 |
| PFI0875w | Pink | 8 | 17 | 0.023 |
| PFF0920c | Grey | 1 | 8 | 0.024 |
| PFB0595w | Pink | 4 | 6 | 0.027 |
| PFI1610c | Pink | 10 | 24 | 0.029 |
| MAL7P1.119 | Grey | 1 | 10 | 0.030 |
| PF14_0499 | Pink | 8 | 18 | 0.034 |
| PFD0080c | Pink | 8 | 18 | 0.034 |
| PF11_0165 | Blue | 1 | 9 | 0.034 |
| PFD0545w | Blue | 1 | 11 | 0.041 |
| *PF13_0197 | Pink | 8 | 19 | 0.047 |
|  |  |  |  |  |
| * Associated with cell invasion | | | |  |
| Number of Cluster Genes in Whole PPI network | | | |  |
|  | Blue (Brain) | 5 |  | NS |
|  | Pink (Lung) | 298 |  | 3.30E-012 |
|  | Orange (Liver) | 1 |  | NS |
|  | Grey (Spleen) | 4 |  | NS |

NS = Non-significant
