# Supplementary material for: Simultaneous host and parasite expression profiling identifies tissue-specific transcriptional programs associated with susceptibility or resistance to experimental cerebral malaria
Source: BMC Genomics. 2006 Nov 22;7:295. doi: 10.1186/1471-2164-7-295 (PMC1664577; doi:10.1186/1471-2164-7-295)
Supplement: Additional file 3 — Correlation between qRT-PCR results and microarray intensity data. R2 values for each gene, comparing qRT-PCR results and microarray intensity data at all time points in all tissues and both mouse strains. [file 1471-2164-7-295-S3.doc]

Table 2: Correlation between qRT-PCR Results and Microarray Intensity Data

| **Alias** | **Description** | **Annotation** | **R^2^** |
| --- | --- | --- | --- |
| ***M. musculus* Genes** | |  |  |
| Ccr4 | Chemokine (C-C motif) receptor 4 | NM_009916 | 0.334 |
| Crp | C-reactive protein, petaxin related | NM_007768 | 0.990 |
| Fcrg2b | Fc Receptor (CD32), IgG low affinity II | NM_010187 | 0.375 |
| Gbp1 | Guanylate nucleotide binding protein 1 | NM_010259 | 0.915 |
| H2-T23 | Histocompatibility 2, T region locus 23 | ENSMUSG00000057787 | 0.713 |
| Hebp1 | Heme binding protein 1 | NM_013546 | 0.854 |
| Icam1 | Intercellular adhesion molecule 1 (CD54) | NM_010493 | 0.911 |
| Ifi204 | Interferon activated gene 204 | NM_008329 | 0.738 |
| Ifi47 | Interferon gamma inducible protein, 47 kDa | NM_010999 | 0.257 |
| Ifit1 | Interferon-induced protein with tetratricopeptide repeats 1 | NM_008331 | 0.760 |
| Ifitm3l | Interferon induced transmembrane protein 3-like | NM_025378 | 0.838 |
| Ifnar2 | Interferon alpha receptor 2 | NM_010509 | 0.666 |
| Ifng | Interferon, gamma | NM_008337 | 0.546 |
| Il18 | Interleukin 18 (IFN-gamma-inducing factor) | NM_008360 | 0.283 |
| Mpa2 | Macrophage activation 2 | NM_008620 | 0.725 |
| nfkb1 | Nuclear factor of kappa light chain gene enhancer in B-cells 1, p105 | NM_008689 | 0.437 |
| oasl1 | 2'-5' oligoadenylate synthetase-like 1 | NM_145209 | 0.714 |
| Oasl2 | 2'-5' oligoadenylate synthetase-like 2 | ENSMUSG00000029561 | 0.383 |
| Pttg | Pituitary tumor-transforming 1 | NM_013917 | 0.623 |
| SamHd | SAM domain and HD domain, 1 | NM_018851 | 0.864 |
| Serpini2 | Serine (or cysteine) proteinase inhibitor, clade I,member 1 | NM_026460 | 0.860 |
| Stat1 | Signal transducer and activator of transcription 1 | NM_009283 | 0.724 |
| Stat2 | Signal transducer and activator of transcription 2 | NM_019963 | 0.623 |
| Tlr4 | Toll-like receptor 4 | ENSMUSG00000039005 | 0.778 |
| Tlr9 | Toll-like receptor 9 | NM_031178 | 0.812 |
| Tnfa | Tumor necrosis factor alpha | NM_013693 | 0.647 |
| Treml4 | Triggering receptor expressed on myeloid cells-like 4 | NM_172623 | 0.805 |
| Vcam1 | Vascular cell adhesion molecule 1 | NM_011693 | 0.486 |
| Vegfa | Vascular endothelial growth factor A | NM_009505 | 0.889 |
|  |  |  |  |
| ***P. berghei* ANKA Genes** | |  |  |
| CSP | Circumsporozoite (CS) protein | PB001026.00.0 (PFC0210c) | 0.649 |
| DOHS | Deoxyhypusine synthase | PB001048.02.0 (PF14_0125) | 0.288 |
| FBA* | Fructose-bisphosphate aldolase | PB000757.02.0 (PF14_0425 ) | 0.777 |
| HP1 | Hypothetical protein | PB000304.03.0 (MAL12P1.406) | 0.346 |
| HP2 | Hypothetical protein | PB000973.02.0 (MAL13P1.161) | 0.520 |
| HP3 | Hypothetical protein | PB000753.03.0 (MAL13P1.240) | 0.025 |
| HP4 | Hypothetical protein | PB000869.03.0 (MAL13P1.78) | 0.401 |
| HP5 | Hypothetical protein | PB300647.00.0 | 0.658 |
| HP6 | Hypothetical protein | PB300738.00.0 | 0.314 |
| HP7 | Hypothetical protein | PB405251.00.0 | 0.192 |
| HP8 | Hypothetical protein | PB000962.00.0 (PF10_0151) | 0.155 |
| HP9 | Hypothetical protein | PB000264.00.0 (PF14_0044) | 0.581 |
| HP10 | Hypothetical protein | PB300759.00.0 (PF14_0060) | 0.638 |
| HP11 | Hypothetical protein | PB300241.00.0 (PFC0910w) | 0.829 |
| HP12 | Hypothetical protein | PB000238.02.0 (PFC0970w) | 0.576 |
| HP13 | Hypothetical protein | PB000428.00.0 (PFD0795w) | 0.838 |
| HSPad | ATP-dependent heat shock protein, putative | PB000740.03.0 (PFI0355c) | 0.539 |
| LAD | Lipoamide dehydrogenase | PB000966.02.0 (MAL12P1.310) | 0.838 |
| SGBP | Small GTP-binding protein | PB000267.00.0 (PFD0810w) | 0.575 |
| STRS* | Seryl-tRNA synthetase | PB000709.02.0 (PF07_0073) | 0.748 |
| TRANS1 | Transporter, putative | PB000201.02.0 (PFE0825w) | 0.199 |
| TRDIPP | tRNA delta(2)-isopentenylpyrophosphate transferase, put. | PB001246.02.0 (MAL12P1.76) | 0.484 |
| * PbA Housekeeping gene | |  |  |
